# The St. Andrew's Institute

**Published:** 1920-01-31

**Authors:** 


### The Hospital

The Workers' Newspaper of Administrative Medicine and Institutional Life, Administration, National Insurance and Health.

No. 1756, Vol. LXVII. SATURDAY, JANUARY 31, 1920. PRICE TWOPENCE

### THE ST. ANDREW'S INSTITUTE.

In the recent address delivered by Sir James Mackenzie at the opening of St. Andrew's Institute for clinical research, and fully reported in the current medical journals, can be found such full and lucid description of the manifold reasons which prompted its establishment, that there is no need for us to attempt further detail here. But neither the results to which this and similar much-needed institutions may lead, nor the essential difference in method by which they attack the research question, have been so clearly defined. The originator himself doubtless brought home the finest shade of meaning in his spoken words, but set down in cold print it is not so easy for the uninitiated immediately to gather up the finer points.

The first consideration that Sir James would have us appreciate is that the surroundings in which research is carried on to-day, in laboratories and hospital wards, in no sense provide the opportunities necessary for discovery of either the early stages, or the predisposing stages of disease. Obviously this does not apply to every malady to which mankind is heir, but it most certainly does to a great majority. There is too often a blank, as it were, between the first infection, the actual onset of the pathological process and that period, typified with regrettable persistence in our great hospitals, when the first gross physical signs provoked by derangement of function appear. We are asked seriously to review the questions as to whether there are not, in many of these cases, symptoms provoked *before* damage is done. Are we sure that the patient's first supposedly indeterminate consciousness of ill-health would not, on closer analysis and better-trained observation, resolve itself into a clinical picture from which information of the greatest value might be gleaned? Therefore does Sir James Mackenzie urge the paramount necessity for this training as an essential factor in successful research, and that such investigation should be carried on in those places where the early stages *can* be seen and the conditions predisposing to disease readily studied. And, as he says, "That, shortly, is the object of our institute and the justification of our enterprise."

The rebuke to the general press which his present address and, to a greater extent, his remarkable book, *The Future of Medicine*, indirectly contain, lies in his clear demonstration of the fact that public attention has recently, persistently but wrongly, been focussed on research from one aspect only, namely the laboratory side of the problem, and the application in medicine of methods proper to a number of allied sciences. We ourselves in these pages have many times outlined and praised the medical triumphs of chemistry, physiology, bacteriology and the like, but at the same time we have insisted on the essentially clinical nature of the problem when dealing with the very beginnings of disease. On the other hand, as a notable contemporary points out, Sir James Mackenzie's proposition may come as a new, perhaps confusing, light even to those medical men who have been brought up to the popular conviction that the highest form of research must be modelled on laboratory lines, with instruments of precision to confirm or correct the impressions and conclusions of the old-time bedside observer. There is an art as well as science in diagnosis, and at no stage of disease is public appreciation of this fact more essential than that which the new Institute is setting out to unravel. Public sympathy and public support must be given to all such schemes, and it is greatly to be hoped that the attempt at St. Andrew's will receive that general appreciation which it deserves and which the press throughout the country may well be advised to cultivate.

Of Sir James Mackenzie himself let us recall that, though an advocate of a fundamental doctrine, in truth so old that to many of the recent school it has appeared almost as a novelty, he was the originator of modern cardiology, mainly through the most careful and accurate instrumental investigation of the circulatory system. A monumental work and one which amply proves the new Director to possess a balanced experience and a firm grasp on the principles of true scientific method which, though deposed from its popular supremacy, is still essential to the work as a whole. In founding the Institute he has made yet another experiment, and one of the greatest significance. Of its success we feel assured, not only in proving the essential truths contained in *The Future of Medicine*, but in providing, to the profession in general, lessons invaluable in their daily care of the people's health.

